# Level of knowledge, stress and acceptance of illness in young adults with type 1 diabetes mellitus

**DOI:** 10.3389/fendo.2025.1648260

**Published:** 2025-09-17

**Authors:** Anna Stefanowicz-Bielska, Małgorzata Rąpała, Kamila Mazuryk, Ewa Dygaszewicz

**Affiliations:** ^1^ Division of Internal and Pediatric Nursing, Institute of Nursing and Midwifery, Faculty of Health Sciences with the Institute of Maritime and Tropical Medicine, Medical University of Gdansk, Gdansk, Poland; ^2^ Department of Paediatrics, Diabetology and Endocrinology, University Clinical Center of Gdansk, Gdansk, Poland; ^3^ Department of Pediatric Surgery, Marciniak Hospital, Wroclaw, Poland; ^4^ Division of Surgical Nursing, Institute of Nursing and Midwifery, Faculty of Health Sciences with the Institute of Maritime and Tropical Medicine, Medical University of Gdansk, Gdansk, Poland; ^5^ Division of Hypertension and Diabetology, University Clinical Center, Gdansk, Poland; ^6^ Voivodeship Branch of Gdansk, Polish Association of Diabetics, Gdansk, Poland

**Keywords:** type 1 diabetes mellitus, knowledge, stress, acceptance, illness, young adult

## Abstract

**Introduction:**

The chronic character of Type 1 Diabetes Mellitus (T1DM) causes patients to be fully aware of the essence and consequences of their illness and to suffer from stigmatisation, tiredness, stress, fear, anxiety and poor mental health.

**Purpose of the paper:**

The purpose of this paper is to assess the level of knowledge, stress and acceptance of the illness in young adults with T1DM and to present the impact of various sociodemographic and medical factors on the level of knowledge, stress and acceptance of the illness in young adults with T1DM.

**Materials and methods:**

This study used an original survey and the psychological PSS-10 and AIS questionnaires among young adults with T1DM who had been ill for more than a year. The survey was conducted from 01.08.2023 to 30.11.2024.

**Results:**

The survey involved 274 young adults aged 18 to 35 years, who had T1DM for 13.4 ± 7.1 years on average. The medians of the test and raw scores for the respondents’ answers to statements in the AIS psychological questionnaire were 18 (17÷19) and 29 (23÷35), respectively, whereas the medians of the raw score and sten scores in the responses given in the PSS-10 psychological questionnaire were 20 (15÷24) and 7 (5÷8), respectively. A negative relationship was confirmed between the level of knowledge and the HbA1c concentration. The survey indicated that women with primary education, being in a relationship, smoking, having hypertension, hypothyroidism and lipohypertrophy, and being treated with multiple daily injections (MDI; automatic insulin pen) had high stress levels. There was a positive relationship between the level of stress experienced by the respondents and their BMI, as well as between the duration of the illness and the number of hyperglycaemic incidents at night. The survey indicated that people with primary education, being single, nonsmoking, not keeping a “paper” self-monitoring journal and having regular nursing and educational appointments at the Diabetes Clinic better accept their illness. The survey confirmed a negative relationship between the level of acceptance of the illness and the HbA1c concentration and hyperglycaemic incidents during the day.

**Conclusions:**

The level of stress experienced by young T1DM patients is high. Young T1DM patients do not accept the illness. Understanding treatment principles helps patients achieve metabolic balance in a significant way. The level of stress, the level of acceptance of the illness doesn’t have relation to the level of knowledge. Contemporary technologies used in T1DM self-monitoring and treatment reduce the level of stress and help patients accept and adapt to the illness. The use of MDI generates a high level of stress in young T1DM patients, and the fact that they do not need to keep a “paper” self-monitoring journal helps them better accept the illness. Educational nurses support young T1DM patients in diabetes therapy and help them accept their illness. Young adults with T1DM need support of psychologists.

## 1 Introduction

Type 1 Diabetes Mellitus (T1DM) is an autoimmune illness that is increasingly common among young people under 20 years of age (1, 2). T1DM complications include acute life-threatening conditions (hypoglycaemia and ketoacidosis) and long-term micro- and macrovascular complications (2, 3).

The destruction of β cells of the pancreas in T1DM patients entails intensive insulin therapy and intensive self-monitoring for life (1, 2). T1DM therapy is based on self-monitoring, i.e., glycaemia monitoring, insulin adjustment, handling hypoglycaemia and hyperglycaemia, counting quantities of carbohydrates consumed by a patient and remembering factors that influence the level of glycaemia, such as physical activity, hormones, stress and temperature. Self-monitoring requires mental maturity, relevant knowledge and skills and frequent decision-making (2, 4, 5). It is essential for T1DM therapy to be efficient, and it is a key element in the process of maintaining a healthy lifestyle and preventing long-term micro- and macrovascular complications (6).

Young adults with T1DM face crucial stages of social maturity in their adult life. They are expected to become fully responsible for their treatment (7).

Chronic T1DM challenges the mental welfare of patients and leads to awareness, stigmatisation, tiredness, stress, fear, poor mental health and negative associations, anxiety, depression and low quality of life (6, 8).

A total of 20%–30% of people with T1DM suffer from stress related to the treatment of diabetes (7).

The purpose of this study was to assess the level of knowledge, stress and acceptance of the illness in young adults with T1DM and to present the impact of various sociodemographic and medical factors on the level of knowledge, stress and acceptance of the illness in young adults with T1DM.

## 2 Materials and methods

### 2.1 Study design, setting and participants

The interview survey was conducted from 01.08.2023 to 30.11.2024.

The participants were young adults with T1DM who had been ill for more than a year, were treated at one of several diabetes clinics in the Pomeranian Voivodeship and were members of the Voivodeship Branch of Gdansk of the Polish Diabetes Association.

The respondents were 18 to 35 years old.

### 2.2 Methods

The cross-sectional observational study was conducted via an original survey and standardised psychological questionnaires: the *Perceived Stress Scale* (PSS-10), developed by S. Cohen, T. Kamarck, R. Mermelstein (adapted to Polish conditions by Z. Juczyński and N. Ogińska-Bulik), and the *Acceptance of Illness Scale* (AIS), developed by B. J. Felton, T. A. Revenson and G. A. Hinrichsen (adapted to Polish conditions by Z. Juczyński) (9, 10).

#### 2.2.1 Original survey form

The survey form contained information about the survey, an invitation to the survey, questions concerning sociodemographic data (gender, age, place of residence, education, marital status, professional activity, school activity, use of addictive substances), medical data related to the illness (concentration of glycosylated haemoglobin (HbA1c) level, duration of the illness, existence of long-term micro- and macrovascular complications, T1DM self-monitoring and treatment methods, regularity of meetings with nurses and doctors) and a knowledge test.

#### 2.2.2 T1DM self-monitoring and treatment test

Knowledge was verified by the use of 20 statements concerning T1DM self-monitoring and treatment, where the respondents were asked to specify whether the statements were correct or incorrect. The respondents were asked about the definition and treatment of T1DM, the effect of insulin and the place of administration, the principles of glycaemia monitoring with a glucometer, risk factors, the symptoms and prevention of hypoglycaemia and hyperglycaemia, the principles of physical activity and types of long-term micro- and macrovascular complications. The knowledge test was prepared on the basis of the *Practical recommendations in nursing and obstetric care in diabetes - 2023. Position of the Polish Federation of Education in the Treatment of Diabetes and Clinical Recommendations for People with Diabetes - 2025. Polish Diabetes Society.* Each correct answer was awarded one point. The maximum score was 20.

#### 2.2.3 Perceived Stress Scale

The PSS-10 is used to measure perceived stress. It contains 10 questions concerning various subjective feelings related to personal affairs and events, ways of conduct, and handling methods. A responder answers by entering a relevant digit, i.e., 0 – never, 1 – almost never, 2 – sometimes, 3 – fairly often and 4 – very often. The internal consistency of the survey was verified in a group of 120 adults, and the Cronbach’s alpha was 0.86. In the original version, the internal reliability of the scale, as assessed on the basis of Cronbach’s alpha, ranged from 0.84 to 0.86 for the three samples studied by Cohen et al. (1983).

Before a general perceived stress intensity ratio is calculated, it is necessary to change the scoring for the answers to positive questions, i.e., 4, 5, 7 and 8, in accordance with the following rule: 0 = 4, 1 = 3, 3 = 1 and 4 = 0. The scores for answers to questions 1, 2, 3, 6, 9 and 10 are not changed. The general scale outcome is the sum of all the scores, whose theoretical distribution ranges from 0 to 40. The greater the score is, the greater the perceived stress. The general ratio, when converted into standardised units, is subject to interpretation adjusted to the characteristics of the standard ten (sten) scale. A score of 1 to 4 sten is treated as low, whereas a score of 7 to 10 sten is high. Scores of 5 and 6 sten are treated as average. The scale result reflects a general assessment of the mental comfort connected with problem handling. A high stress perception ratio on the PSS-10 is associated with various mental and somatic symptoms, which is why it is a measure of chronic stress, which is a risk factor for various illnesses (9).

With the consent of the Psychological Test Laboratory of the Polish Psychological Society, the paper version of the PSS-10 was used.

#### 2.2.4 Acceptance of Illness Scale

The AIS consists of eight statements describing the negative consequences of poor health. Those consequences include the acceptance of limitations resulting from the illness, the lack of self-sufficiency, the feeling of dependence on other people, and reduced self-esteem. Acceptance of the illness contributes to a smaller intensity of negative relationships and emotions connected with the illness.

The scale is used to measure the level of acceptance of an illness. The greater the acceptance, the better the adaptation and the smaller the sense of mental discomfort. The scale may be used to assess the level of acceptance of any illness.

All the AIS statements reflect the specific difficulties and limitations caused by the illness. Strong agreement (grade 1) reflects poor adjustment to the illness, and strong disagreement (grade 5) reflects acceptance of the illness. Thus, the total score is a general measure of the acceptance of the illness and comes within 8 to 40. A low score indicates a lack of acceptance of and adaptation to the illness, as well as a strong sense of mental discomfort. In turn, a high score reflects the acceptance of one’s own health condition, which means the absence of negative emotions related to the illness (10).

### 2.3 Data collection

The researchers distributed the survey forms and psychological questionnaires to the participants in person. Participation in the survey was voluntary. The survey was approved by the Management of Health care Institutions and the President of the Voivodeship Branch of Gdansk of the Polish Diabetes Association in the Pomeranian Voivodeship, as well as by the Independent Bioethical Commission for Scientific Studies at the Medical University of Gdansk (KB/351/2023).

### 2.4 Description of the statistical methods

The results were subjected to statistical processing. The number of cases (N), mean, standard deviation (SD), median, range (min–max), and lower and upper quartiles (25Q–75Q) of the quantitative parameters were calculated for all groups.

Depending on the distribution, the following quantitative data were presented:

1. as the mean ± SD, in the case of variables with normal distribution;
2. as the median and interquartile range M (25Q÷75Q), in the case of variables with nonnormal distribution.

Qualitative variables are presented as absolute values and percentages (%).

The normality of the distribution was tested with the Shapiro–Wilk test, and the homogeneity of variance was checked with Levene’s test.

Verification of the hypothesis of equality of mean parameters in independent groups with homogeneous variance was performed by one-way analysis of variance (ANOVA) or for groups with heterogeneous variance via the nonparametric Mann–Whitney U test (for two groups) and the Kruskal–Wallis test (for three or more groups).

The relationship between two parameters was assessed using correlation analysis, and Spearman correlation coefficients (R) were calculated.

A p value of less than 0.05 was required to reject the null hypothesis. Statistical analysis was performed using the computer statistical software package Statistica Ver. 13.3. (TIBCO Software Inc.).

## 3 Results

### 3.1 Description of the participants

A total of 274 young adults with T1DM, aged 18-35 years, participated in the survey. Most of the respondents were women. Participants had T1DM for 13.4 ± 7.1 years on average, were treated by the use of with a personal insulin pump, had secondary education, lived in a large town, were in an informal relationship, were professionally active, were not learning at school, and did not smoke or consume alcohol. The average Body Mass Index (BMI) and the HbA1c concentration were 25 ± 4.7 kg/m^2^ and 7.94 ± 1.68%, respectively. The median time in range (TIR) of the respondents was 70 (50÷70). Characteristic of the participants shows **Table 1**.

**Table 1:** Sociodemographic data vs. the level of knowledge, acceptance of illness and stress in young adults with T1DM.

Factor		N (%)	Level of knowledge	Level of acceptance of the illness - raw score	Level of stress - raw score	Level of stress - sten
gender	female	163 (59.5%)	19(18÷20)	27.6 ± 8.3	20.5 ± 6.5	6.73 ± 1.90
	male	111 (40.5%)	18(17÷19)	29.6 ± 8.2	18.4 ± 6.2	6.16 ± 1.83
p			0.00047**	0.0516*	0.00775*	0.0143*
age (years)		274 (100%)	26 ± 5			
HbA1c level (%)		274 (100%)	7.94 ± 1.68			
TIR (%)		81 (29.6%)	71 (50÷70)			
weight (kg)		274 (100%)	74.5 ± 16.1			
BMI (kg/m^2^)		274 (100%)	25 ± 4.7			
duration of illness (years)		274 (100%)	13.4 ± 7.1			
number of incidents of hypoglycaemia during the day		274 (100%)	1(0÷2)			
number of incidents of hypoglycaemia at night		274 (100%)	1(1÷2)			
number of incidents of hyperglycaemia during the day		274 (100%)	1(1÷2)			
number of incidents of hyperglycaemia at night		274 (100%)	1(1÷1)			
place of residence	small town	45 (16.4%)	19(18÷20)	29(23÷32)	18(14÷23)	6(5÷8)
	medium town	75 (27.4%)	18(17÷19)	28(22÷34)	21(18÷25)	7(6÷8)
	large town	98 (35.8%)	18(17÷19)	30.5(24÷36)	20(14÷24)	7(5÷8)
	village	56 (20.4%)	18(18÷19)	27(22÷37)	18(13÷24)	6(4÷8)
p			0.0896***	0.762***	0.0775***	0.0784***
education	primary	19 (6.9%)	18(17÷19)	32(28÷35)	25(19÷28)	8(6÷9)
	secondary	121(44.2%)	18(17÷19)	29(20÷34)	20(15÷24)	7(5÷8)
	vocational	45(16.4%)	19(18÷20)	23(21÷31)	19(16÷23)	6(5÷8)
	tertiary	89(32.5%)	18(17÷19)	32(25÷37)	19(15÷24)	6(5÷8)
p			0.735***	0.0020*** vocational vs tertiary p=0.003	0.0357*** primary vs secondary p=0.0326 primary vs tertiary p=0.031	0.0503*** primary vs tertiary p=0.0479
marital status	married	74(27%)	18.2 ± 2.1	28.4 ± 7.3	21.5(18÷24)	7(6÷8)
	informal relationship	107(39%)	17.5 ± 3.2	26.7 ± 8.7	20(16÷24)	7(5÷8)
	single	93(34%)	17.5 ± 3.1	30.3 ± 8.2	18(13÷24)	6(4÷8)
p			0.226*	0.00935* informal relationship vs single p=0.009	0.0780***	0.0253*** married vs single p=0.0238
professionally active	yes	192(79.7%)	17.6 ± 2.9	28.2 ± 8.1	19.8 ± 6.2	6.57 ± 1.82
	no	49(20.3%)	17.5 ± 3.7	29.5 ± 7.5	19.7 ± 6.6	6.43 ± 2
p			0.833*	0.309*	0.927*	0.640*
continuation of school education	yes	86(31.4%)	18.1 ± 2.6	28.8 ± 8.3	18.7 ± 6.9	6.14 ± 2.02
	no	188(68.6%)	17.5 ± 3	28.2 ± 8.3	20.1 ± 6.2	6.66 ± 1.81
p			0.129*	0.612*	0.0998*	0.0324*
smoking	yes	77(28.1%)	18(16÷19)	26.7 ± 8.2	21(18÷24)	7(6÷8)
	no	197(71.9%)	19(18÷19)	29 ± 8.2	19(14÷24)	6(5÷8)
p			0.00230**	0.0343*	0.0210**	0.0147**
alcohol consumption	yes	184(67.2%)	19(17÷19)	29 (23÷34)	19.9 ± 6.3	6.60 ± 1.86
	no	90(32.8%)	18(18÷19)	31 (21÷38)	19.2 ± 6.7	6.30 ± 1.95
p			0.804**	0.0775**	0.413*	0.221*
regular meetings with a doctor at the Diabetes Clinic	yes	226(82.5%)	19(18÷19)	29 (23÷36)	20(15÷25)	7(5÷8)
	no	48(17.5%)	18(14÷19)	28 (23÷32)	20(18÷23)	7(6÷8)
			0.00065**	0.0966**	0.894**	0.654**
regular nursing and educational meetings at the Diabetes Clinic	yes	61(22.3%)	17.9 ± 2.5	30.9 ± 7.1	18.9 ± 6.7	6.25 ± 1.85
	no	213(77.7%)	17.6 ± 3	27.7 ± 8.5	19.9 ± 6.4	6.57 ± 1.85
p			0.467*	0.00751*	0.270*	0.234*

*ANOVA; **Mann–Whitney U test; ***Kruskal–Wallis test.

### 3.2 T1DM self-monitoring and treatment test

The median score of the test was 18 (17÷19), with a maximum score of 20 and a minimum score of 1. The following statements were the most difficult for the respondents: Statement 2. *(Type 1 diabetes develops as a result of excessive sugar consumption, overweight, obesity and a lack of physical activity):* 75.2% of correct statements; Statement 9. (*The level of glycaemia (glucose concentration) in blood that is correct for an adult with type 1 diabetes is from 80 to 140.):* 78.1% of correct statements; Statement 15. (*Hyperglycaemia is diagnosed if the level of glycaemia (glucose) in the blood is ≥ 160–180 mg/dl (mg%)):* 79.2% of correct statements; and Statement 17. (*Heavy hypoglycaemia is a contraindication to physical activity for 24 hours*): 70.1% of correct statements (**Table 2**).

**Table 2:** Results of the test on T1DM self-monitoring and treatment in young adults with T1DM.

No	Statement	True		Not true	
		N	%	N	%
1	T1DM is a chronic illness.	242	88.3	32	11.7
2	T1DM develops as a result of excessive sugar consumption, overweight, obesity and a lack of physical activity.	206	75.2	68	24.8
3	T1DM treatment involves correct nutrition, physical activity, insulin therapy and regular self-monitoring.	256	93.4	18	6.6
4	Insulin reduces the level of glycaemia.	259	94.5	15	5.5
5	Insulin is injected in body areas that are rich in loose subcutaneous tissue (abdomen, thigh, buttocks, arm).	253	92.3	21	7.7
6	Insulin must not be injected in the local fat tissue within the area of the insulin injection place (lipohypertrophy).	228	83.2	46	16.8
7	A T1DM patient must measure the level of glycaemia regularly.	259	94.5	15	5.5
8	Prior to taking capillary blood to measure the level of glycaemia with a glucometer, you are recommended to wash your hands carefully with warm water and soap without disinfection agents and then dry them thoroughly.	259	94.5	15	5.5
9	The level of glycaemia in blood that is correct for a T1DM patient is from 80 to 140 mg/dl (mg%).	214	78.1	60	21.9
10	Hyperglycaemia is diagnosed if the level of glycaemia in blood is ≤ 70 mg/dl (mg%).	254	92.7	20	7.3
11	Risk factors for hypoglycaemia include: an excessive dose of insulin, a skipped meal, a reduced portion of carbohydrates, too intensive unplanned physical activity, alcohol consumption and aiming at the fast normalisation of glycaemia in blood.	259	94.5	15	5.5
12	Symptoms of light or medium hypoglycaemia include: worse mood, weakness, tiredness, concentration problems, hyperactivity, behaviour that is not typical for the patient, sudden change in mood, illogical answers to questions, a strong sense of hunger, paleness, excessive sweating, shaky hands, headaches, stomach aches, yawning and drowsiness.	252	92.0	22	8.0
13	During the symptoms of light or medium hypoglycaemia, the T1DM patient should consume 10 g of easily assimilable carbohydrates, e.g. 100 ml of juice or 10 g of glucose in the form of gel or tablets.	244	89.1	30	10.9
14	Glucagon increases the level of glycaemia in blood.	253	92.3	21	7.7
15	Hyperglycaemia is diagnosed if the level of glycaemia in blood is ≥ 160–180 mg/dl (mg%).	217	79.2	57	20.8
16	If, for a longer period, a T1DM patient has a high level of glucose in blood, they will have such symptoms as: worse mood, intensive thirst, a frequent need to pass urine, annoyance, weakness, stomach aches, nausea and vomiting.	259	94.5	15	5.5
17	Heavy hypoglycaemia is a contraindication to physical activity for 24 hours.	192	70.1	82	29.9
18	T1DM patients should have physical activity regularly, at least every 2 to 3 days or even better, every day.	257	93.8	17	6.2
19	Hypoglycaemia and hyperglycaemia are sudden, acute diabetes complications.	224	81.8	50	18.2
20	Nephropathy, retinopathy and neuropathy are remote chronic complications.	252	92.0	22	8

The survey indicated that women, patients without a diagnosis of hypothyroidism, patients who did not smoke, or patients who had regular appointments at the Diabetes Clinic had a better level of knowledge (**Tables 1**, **3**). A negative relationship was confirmed between the level of knowledge and the HbA1c concentration (n=274, R=-0.14, p=0.0241).

**Table 3:** Accompanying illnesses vs. the level of knowledge, acceptance of the illness and stress in young adults with T1DM.

Factor		N (%)	Level of knowledge	Level of acceptance of the illness – raw score	Level of stress - raw score	Level of stress -sten
hypertension	yes	37 (13.5%)	17.5 ± 3.1	27 ± 8.8	22.5 ± 5.8	7.35 ± 1.64
	no	237 (86.5%)	17.7 ± 2.9	28.6 ± 8.2	19.2 ± 6.4	6.37 ± 1.89
p			0.654*	0.267*	0.00326*	0.00305*
hypothyroidism	yes	88 (32.1%)	18(16÷19)	26.8 ± 8	21(18÷24)	7(6÷8)
	no	186 (67.9%)	19(18÷20)	29.1 ± 8.3	19(13÷24)	6(4÷8)
p			0.00545**	0.0280*	0.0127**	0.0100**
hyperthyroidism	yes	12 (4.4%)	18.5(16.5÷19)	27(18÷33)	21.5(19÷26)	7(6÷8.5)
	no	262 (95.6%)	18(17÷19)	29(23÷35)	20(15÷24)	7(5÷8)
p			0.535**	0.219**	0.0112**	0.173**
coeliac disease	yes	19 (7%)	17(10÷19)	24(22÷31)	20(17÷25)	7(6÷8)
	no	255 (93%)	18(18÷19)	29(23÷35)	20(15÷24)	7(5÷8)
p			0.0183**	0.121**	0.620**	0.732**
asthma	yes	12 (4.4%)	18(18÷19)	28(23.5÷29.5)	24(20.5÷28)	8(7÷9)
	no	262 (95.6%)	18(17÷19)	29(23÷35)	20(15÷24)	7(5÷8)
p			0.908**	0.228**	0.02**	0.01**
rheumatoid arthritis	yes	8 (3%)	19.5(19÷20)	21(15.5÷28)	33(19.5÷33)	10(6.5÷10)
	no	266 (97%)	18(17÷19)	29(23÷35)	20(15÷24)	7(5÷8)
p			0.0379**	0.0416**	0.0112**	0.0126**
diabetic nephropathy	yes	7 (2.5%)	18(18÷20)	17(12÷36)	21(16÷29)	7(5÷9)
	no	267 (97.5%)	18(17÷19)	29(23÷35)	20(15÷24)	7(5÷8)
p			0.674**	0.0619**	0422**	0.483**
diabetic retinopathy	yes	10 (3.6%)	18(18÷19)	12.5(12÷31)	23.5(20÷27)	7.5(7÷9)
	no	264 (96.4%)	18(17÷19)	29(23÷35)	20(15÷24)	7(5÷8)
p			0.533**	0.0161**	0.0302**	0.0717**
diabetic neuropathy	yes	15 (5.5%)	19(18÷20)	25(12÷30)	20(10÷25)	7(4÷8)
	no	259 (4.5%)	18(17÷19)	29(23÷35)	20(16÷24)	7(5÷8)
p			0.0974**	0.0960**	0.942**	0.992**
lipohypertrophy	yes	53 (19.3%)	19(18÷19)	27.3 ± 8.2	21.4 ± 5.7	7(6÷8)
	no	221 (80.7%)	18(17÷19)	28.6 ± 8.3	19.2 ± 6.5	6(5÷8)
p			0.667**	0.292*	0.0251*	0.0392**
lipoatrophy	yes	6 (2.2%)	18(14÷18)	18(16÷24)	20.5(16÷22)	7(5÷7)
	no	268 (97.8%)	18(17÷19)	29(23÷35)	20(15÷24)	7(5÷8)
p			0.0646**	0.0266**	0649**	0.682**

*ANOVA; **Mann–Whitney U test; ***Kruskal–Wallis test.

### 3.3 Perceived Stress Scale

The respondents’ scores on the Perceived Stress Scale were analysed.

The median raw score of the responses given in the PSS-10 was 20 (15÷24) and the median sten score was 7 (5÷8).

The survey indicated that women with primary education, being in a relationship, smoking, having hypertension, hypothyroidism and lipohypertrophy and being treated with multiple daily injections (MDI; automatic insulin pen) had high stress levels (**Tables 1**, **3**, **4**).

**Table 4:** T1DM self-monitoring and treatment method vs. level of knowledge, acceptance of the illness and stress in young adults with T1DM.

Treatment method		N (%)	Level of knowledge	Level of acceptance of the illness - raw score	Level of stress - raw score	Level of stress - sten
automatic insulin pen	yes	126 (46%)	18(17÷19)	28 ± 7.8	20.5(18÷25)	7(6÷8)
	no	148 (54%)	18.5(17÷19)	28.7 ± 8.7	19(13.5÷24)	6(4.5÷8)
p			0.725**	0.503*	0.0428**	0.0392**
“smart” insulin pen	yes	10 (3.6%)	18.5(16÷20)	31.5(21÷36)	19.5(16÷25)	8(6.5÷6.5)
	no	264 (96.4%)	18(17÷19)	29(23÷35)	20(15÷24)	7(5÷8)
p			0.900**	0.874**	0.982**	0.966**
personal tubed insulin pump	yes	132 (48.2%)	17.8 ± 3.1	28.9 ± 8.7	19(13.5÷24)	6.3 ± 1.95
	no	142 (51.8%)	17.5 ± 2.7	27.9 ± 7.9	20(17÷25)	6.68 ± 1.82
p			0.391*	0.275*	0.0543**	0.0962*
use of bolus calculator in personal tubed insulin pump	yes	116 (42.3%)	17.8 ± 3.3	29.1 ± 8.6	19(13÷24)	6.28 ± 1.98
	no	158 (57.7%)	17.5 ± 2.6	27.9 ± 8	20(17÷25)	6.66 ± 1.81
p			0.549*	0.244*	0.0673**	0.106*
personal tubeless insulin pump (insulin patch pump)	yes	7 (2.6%)	13(10÷17)	25(19÷34)	22(17÷25)	7(6÷8)
	no	267 (97.4%)	18(17÷19)	29(23÷35)	20(15÷24)	7(5÷8)
p			0.00038**	0.334**	0.592**	0.522**
use of bolus calculator in personal tubeless insulin pump (insulin patch pump)	yes	8 (2.9%)	15(10.5÷17.5)	23.5(18.5÷31)	21.5(17.5÷24)	7(6÷8)
	no	266 (97.1%)	18.5(17÷19)	29(23÷35)	20(15÷24)	7(5÷8)
p			0.00064**	0.161**	0.552**	0.503**
glucometer	yes	105 (38.3%)	18(17÷20)	29(24÷34)	20(16÷24)	7(5÷8)
	no	169 (61.7%)	18(17÷19)	29(22÷36)	20(14÷24)	7(5÷8)
p			0.546**	0.897**	0.435**	0.528**
continuous glycaemia monitoring system	yes	159 (58.2%)	19(18÷19)	28.8 ± 8	19(14÷24)	6(5÷8)
	no	114 (41.8%)	18(17÷19)	27.8 ± 8.6	20.5(16÷24)	7(5÷8)
p			0.277**	0.321*	0.161**	0.191**
“traditional” paper self-monitoring journal	yes	32 (11.7%)	17.4 ± 3.1	25.5 ± 7.9	21 ± 5.5	6.94 ± 1.68
	no	241 (88.3%)	17.7 ± 2.9	28.8 ± 8.3	19.5 ± 6.6	6.44 ± 1.91
p			0.650*	0.0331*	0.199*	0.163*
“electronic” self-monitoring journal	yes	84 (30.6%)	18(18÷19)	29.4 ± 8.5	19(13÷25)	6(4÷8)
	no	190 (69.4%)	19(17÷19)	27.9 ± 8.2	20(16÷24)	7(5÷8)
p			0.274**	0.194*	0.720**	0.722**
use of a mobile application to calculate grams of carbohydrates or carbohydrate exchanges	yes	100 (36.5%)	18(18÷20)	28.8 ± 8.3	20.3 ± 6.8	6.60 ± 2.01
	no	174 (63.5%)	18(17÷19)	28.1 ± 8.3	19.3 ± 6.2	6.44 ± 1.82
p			0.161**	0.486*	0.233*	0.508*

*ANOVA; **Mann–Whitney U test; ***Kruskal–Wallis test.

**Figure 1:**
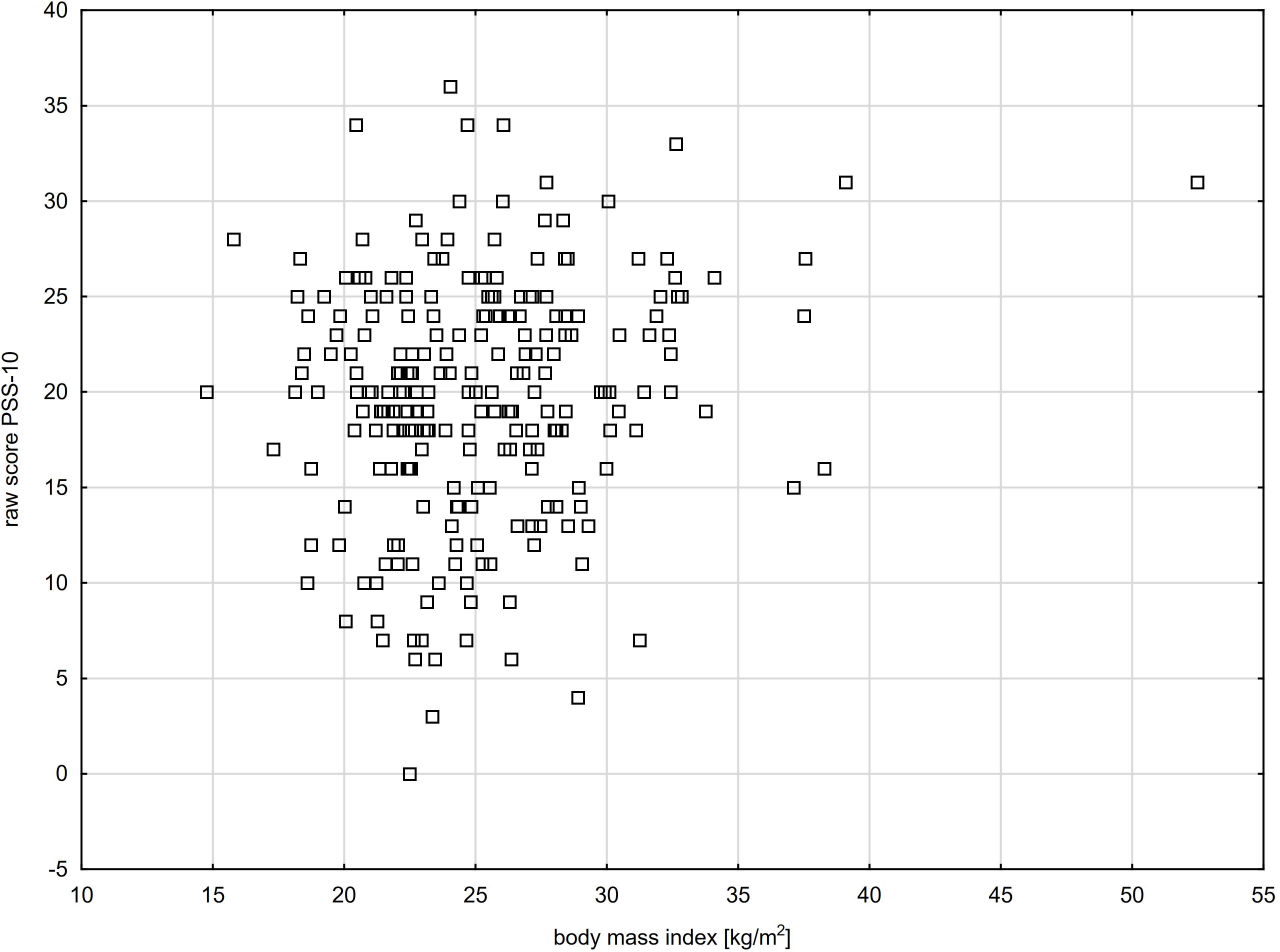
Level of stress experienced by young adults with T1DM in relation to the respondents’ body mass index (R= 0.18, p=0.00275).

There was a positive relationship between the level of stress experienced by the respondents and their BMI (raw score: n=274, R = 0.18, p=0.00275 - **Figure 1**; sten scores: n=274, R = 0.19, p=0.00192), as well as between the duration of illness (raw score: n=274, R = 0.12, p=0.0465; sten scores: n=274, R = 0.11, p=0.0581) and hyperglycaemic incidents at night (raw score: n=217, R = 0.16, p=0.0181; sten scores: n=217, R = 0.15, p=0.0270)). No relationship between the level of stress and the level of knowledge was found (raw score: n=274, R=-0.04, p=0.559; sten scores: n=274, R=-0.03, p=0.569).

### 3.4 Acceptance of Illness Scale

The respondents’ scores on the *Acceptance of Illness Scale* were analysed. The median raw score in the responses given in the AIS psychological questionnaire was 29 (23÷35).

The survey indicated that people with primary education, being single, having hypothyroidism, nonsmoking, not keeping a “paper” self-monitoring journal and having regular nursing and educational appointments at the Diabetes Clinic better accept their illness (**Tables 1**, **3**, **4**).

**Figure 2:**
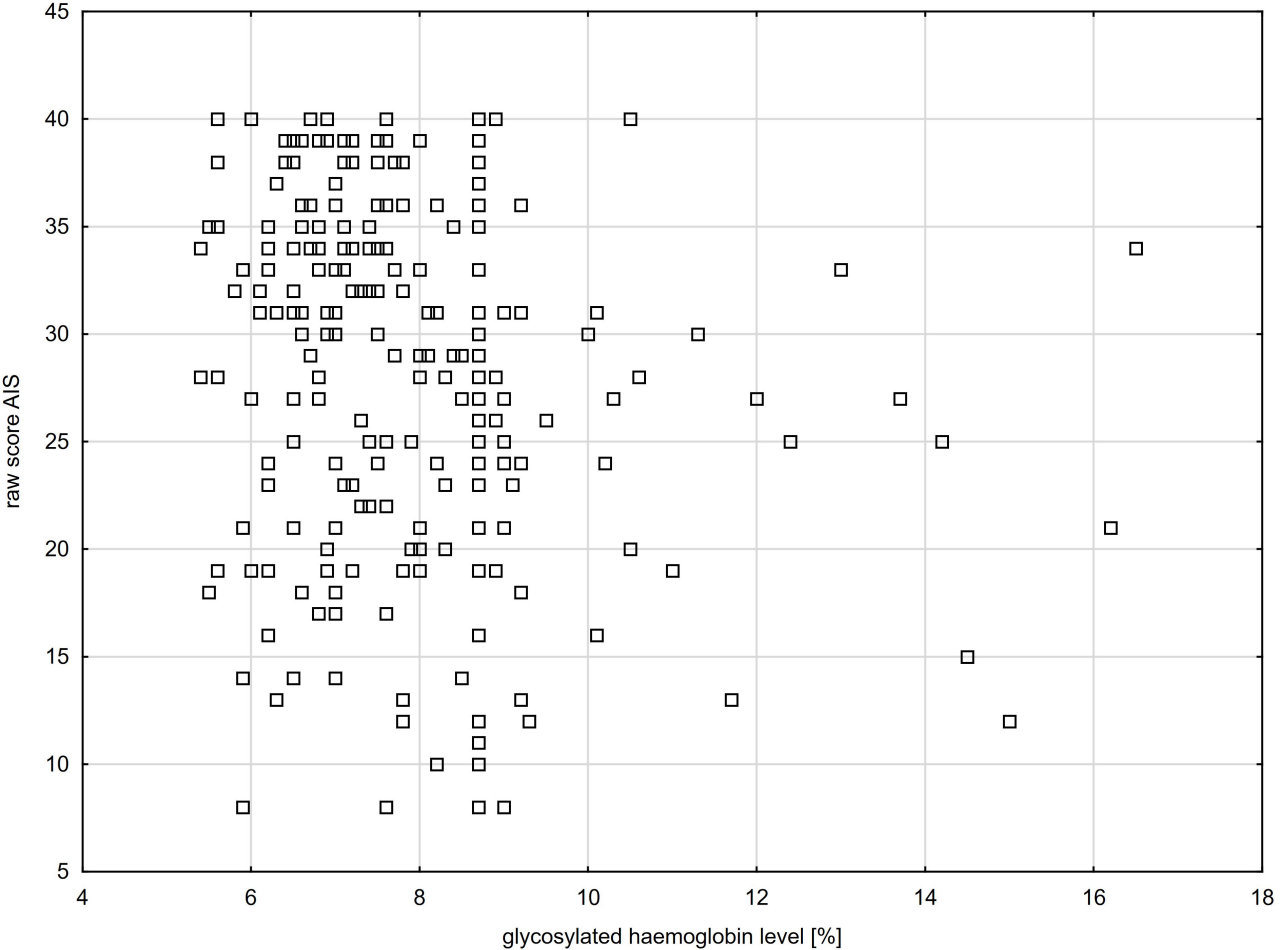
Level of acceptance of the illness in young adults with T1DM in relation to the respondents’ glycosylated haemoglobin level (R= - 0.12, p=0.0473).

The survey confirmed a negative relationship between the level of acceptance of the illness and the HbA1c concentration (raw score: n=274, R=-0.12, p=0.0473 - **Figure 2**) and hyperglycaemic incidents during the day (raw score: n=218, R=-0.14, p=0.0360). No relationship between the level of acceptance of the illness and the level of knowledge was found (raw score: n=274, R = 0.02, p=0.729).

## 4 Discussion

Type 1 diabetes is a chronic incurable illness that influences various aspects of a patient’s life. The diagnosis is usually unexpected and sudden.

Diabetes education is a key element of effective treatment (11–14). Understanding T1DM self-monitoring and treatment principles and efficient T1DM self-management by young people with T1DM reduce the risk of long-term micro- and macrovascular complications, improve treatment results, and positively affect quality of life (15).

The purpose of this study was to assess the level of knowledge, stress and acceptance of the illness in young adults with T1DM and to present the impact of various sociodemographic and medical factors on the level of knowledge, stress and acceptance of the illness in young adults with T1DM.

In our survey a poor relationship between the level of knowledge and the HbA1c concentration was confirmed. On the basis of our survey, understanding the essence of self-monitoring and treatment principles may help achieve metabolic balance in a significant way.

In the comparison with another study differences between HbA1c level and level of knowledge wasn’t found.

In a Brazilian survey, the relationships between glycaemia monitoring and knowledge of diabetes, stress resistance, depression and anxiety were tested in 85 Brazilian teenagers and young adults with T1DM who were 11–22 years of age (17.7 ± 3.72). The researchers used the Resilience Scale (RS), the Hospital Anxiety and Depression Scale (HADS) and the Diabetes Knowledge Assessment Scale (DKNA). The level of glycaemia monitoring was assessed on the basis of HbA1c. The level of HbA1c was 9.3 ± 2.3%. The Brazilian authors reported correlations between the HbA1c concentration and stress resistance (*r=−0.22, p=0.048*), anxiety (*r=0.25, p=0.022*) and depression (*r=0.33, p=0.002*). In turn, there was no correlation between HbA1c and knowledge of diabetes (*r=−0.06, p=0.600*) (16). I think that these discrepancies depend on the number of participants and maybe nationality.

Mansour-Ghanaei et al. assessed 92 patients with T1DM in northern Iran for the relationships among knowledge, illness monitoring, health-related beliefs, the level of HbA1c and the number of attending in health center. The authors did not find a significant relationship between sex, age, marital status, education, profession, duration of illness, place of residence or family history of the patient and their knowledge, health-related beliefs or illness monitoring level (p>0.05). Most respondents had a low level of knowledge (59.8%), poor health-related beliefs (71.7%), and poor self-monitoring (62%). The authors did not find a significant relationship between patients’ knowledge, health-related beliefs or self-monitoring levels and the level of HbA1c (17).

Korean researchers tested educational needs in 100 patients with T1DM (age: 21.85 ± 5.48) and their 93 parents (age: 45.83 ± 4.25). The greatest educational needs included information about the illness, the function of the pancreas and insulin effects, relevant diabetes handling, the role of physical activity, the treatment of complications and advances in diabetes treatment. Educational needs concerning risk factors (p=0.028) and the treatment of complications (p*=*0.021), the performance of physical activity (p*=*0.034), the treatment of diabetes (p*=*0.005), diet (p*=*0.035) and psychosocial needs (p*=*0.001) were greater in the T1DM patients’ carers than in the T1DM patients themselves (p<0.05) (18).

The sudden and unexpected diagnosis of an incurable illness generates many emotions and may also trigger various approaches. Any chronic and incurable illness is a source of stress and influences the process of adaptation to treatment-related principles and limitations. “Diabetes” stress, i.e., stress related to diabetes, involves emotional burdens, stressors and frustration resulting from the need to handle diabetes, which is a serious illness, as well as from illness self-monitoring, which forms the basis of diabetes treatment (19). Our survey indicated that patients treated with MDI had a high stress level. Probably it is connected with number of injections and feeling of pain. The young adults with T1DM need very often psychologist care. The psychologist help is necessary for all patients, especially with high level of stress.

On the basis of our survey, we discovered that the medians of the raw scores and sten scores in the respondents’ answers to the statements in the PSS-10 psychological questionnaire, where the level of stress was assessed, were 20 (15÷24) and 7 (5÷8), respectively. It is interesting and worthy of attention that 25Q of stress level was relatively low. It is difficult to assess the reasons for this. It was proven that women with a primary education and treated with MDI had high stress levels.

Cyranka et al. assessed, among other factors, the level of stress and acceptance of the illness in 50 young patients (n=28.56% women) with T1DM who came to the adult clinic during their first appointment. The average age of the patients was 19.2 ± 1.4 years, the average duration of diabetes was 9.8 ± 4.3 years, the average BMI was 23.5 ± 3.1 kg/m^2^ and the average level of HbA1c was 7.5% ± 1.2%. Sixty-eight percent of the patients were treated with continuous subcutaneous insulin infusion (CSII), and 32% were treated with MDI. The general level of stress in the analysed group was 18.3 ± 7.3 (raw score) and 6.1 ± 2.1 (sten score). In this group, men over 18 years of age reported increased anxiety. The treatment method was not found to have an effect on the level of stress or acceptance of the illness (20).

Another Polish survey assessed the levels of anxiety and depression using the HADS scale in 101 T1DM patients (age: 33.8 ± 13.2 years) and 90 patients with type 2 diabetes mellitus (T2DM) (age: 59.8 ± 6.6 years). The duration of illness in T1DM patients was 17.01 ± 10.98 years. The concentration of HbA1c in T1DM patients was 7.93 ± 1.5%. The average depression score for T1DM patients was 3.93 ± 3.49, and the average anxiety score was 6.88 ± 4.51 (21).

Lunkenheimer et al. examined the relationship between the diagnosis of posttraumatic stress disorder (PTSD) and parameters related to diabetes monitoring in T1DM patients. Patients with T1DM and PTSD (n=179) were compared with a group of T1DM patients without PTSD (n=895) and a group of T1DM patients without accompanying mental disorders (n=895). Patients with PTSD who were ≤25 years of age, in comparison with patients without PTSD or patients without mental disorders, had a significantly greater level of HbA1c (8.71% in comparison with 8.3% or 8.24%), a greater number of hospitalisations (0.94 in comparison with 0.44 or 0.32 per annum) and greater diabetes ketoacidosis (0.1 in comparison with 0.02 or 0.01 incidents/year). Compared with patients without PTSD, patients with PTSD who were ≤25 years of age had a much greater BMI (0.85 in comparison with 0.59). The authors noted that it is necessary to provide psychological support to T1DM patients (22).

In our survey, most patients had been diagnosed with T1DM 13.4 ± 7.1 years prior, on average. The average BMI and HbA1c concentration were 25 ± 4.7 kg/m^2^ and 7.94 ± 1.68%, respectively. We found a positive relationship between the level of stress experienced by the respondents and their BMI, the duration of the illness and hyperglycaemic incidents at night.

Irish researchers attempted to identify the reasons for diabetes stress in 35 young adults with T1DM aged 25–30 years. They reported that diabetes-related stress is common in young adults with T1DM at the second phase of early adulthood (23–30 years of age). Diabetes-related stress is caused by many factors, such as self-awareness/stigmatisation, everyday difficulties related to diabetes treatment, the need to contend with the health care system, fear of the future and fear concerning their future family and pregnancy. The ability to have a conversation with health care practitioners, attend diabetes education programmes and take an active part in support groups mitigated stress in young adults with T1DM. The young adults believed that a conversation with health care practitioners on frustration, stress and difficulties related to diabetes self-monitoring and treatment should be included in the standard diabetes health care system.

Our surveys indicated that no more than 22.3% of the respondents had regular appointments at nursing and educational centres. In turn, 82.5% of the respondents had regular appointments with a doctor at the Diabetes Clinic. We found that the respondents who had regular appointments with a doctor at the Diabetes Clinic had a better level of knowledge. In turn, the respondents who had regular nursing and educational appointments at the Diabetes Clinic better accepted their illness.

Emotional support for T1DM patients during their lengthy treatment helps them accept the diagnosis, strengthens their motivation, and promotes their self-reliance and self-determination.

Taking any active steps usually involves prior acceptance of the illness. If the patient accepts the illness, they take a positive approach to the situation, become aware of the meaning of their illness, mobilise themselves, and adapt more easily.

The acceptance of the illness involves various emotions, which appear during the process of becoming aware of the meaning of the illness itself, as well as during its course and consequences. Feelings such as anger, anxiety and depression with a lack of acceptance of the new situation may lead to chronic depression and long-lasting anxiety (21).

Acceptance prevents a decrease in quality of life and reduces the risk of complications related to the illness. It is very important during the development of a different way of life, including new rules and limitations (21).

In our survey, the median of the raw score in the responses given in the AIS psychological questionnaire was 29 (23÷35). The survey confirmed a negative relationship between the level of acceptance of the illness and the HbA1c concentration and hyperglycaemic incidents during the day. In addition, the survey indicated that people with primary education, being single, not keeping a “paper” self-monitoring journal and having regular nursing and educational appointments at the Diabetes Clinic better accept their illness.

Brzoza et al. assessed the level of acceptance of the illness and the relationship between the illness and quality of life in 101 T1DM patients (50.5% women; age of the respondents: 33.8 ± 13.2) and 90 T2DM patients (55.5% women; age of the respondents: 59.8 ± 6.6). In the AIS, T1DM patients scored 29.56 ± 7.33 on average, whereas T2DM patients scored 31.57 ± 6.25 on average. In T1DM patients, acceptance of the illness was negatively correlated with the intensity of anxiety (hospital anxiety and depression scale - anxiety subscale, HADS-A; r=-0.398, p=0.00003) and depression (hospital anxiety and depression scale – depression subscale, HADS-D; r=-0.390, p=0.00005) and positively correlated with metabolic monitoring (HbA1c; r=-0.259, p=0.008) (21).

In their survey, which involved 50 young T1DM patients (n=28.56% women) visiting the adult clinic for the first time, Cyranka et al. reported that the level of acceptance of the illness was 30.7 ± 7.5 (raw score). The survey indicated a difference in the level of acceptance between the general healthy population and T1DM patients (general healthy population vs. T1DM patients=24.81 ± 7.09 vs. 30.72 ± 7.48, p ≤ 0.001). The treatment method was not found to have an effect on the acceptance of the illness (20).

Acceptance of the illness may be important during the development of a different way of life, including new rules and limitations. There are different approaches to treating this illness. With respect to children or young people suffering from T1DM, their rebellious behaviour influences the life of the whole family. When a child is diagnosed with T1DM, parents “lose” a healthy child and have a chronically ill child who needs permanent care. In turn, the child must adapt their whole life and future plans to self-monitoring rules and treatment methods. The process of acceptance and adaptation is usually long, consists of many stages, and is dependent on various individual factors, such as age, gender, character, ability to handle critical situations and the course of the illness (21).

The main limitation of the study is the small number of participants who were recruited from only two centers, as well as the implementation of the study at a single time point and the lack of a follow-up study.

## 5 Conclusions

The level of stress experienced by young T1DM patients is high. Young T1DM patients do not accept the illness.

Understanding treatment principles helps patients achieve metabolic balance in a significant way.

The level of stress, the level of acceptance of the illness doesn’t have relation to the level of knowledge.

Contemporary technologies used in T1DM self-monitoring and treatment reduce the level of stress and help patients accept and adapt to the illness. The use of MDI generates a high level of stress in young T1DM patients, and the fact that they do not need to keep a “paper” self-monitoring journal helps them better accept the illness. Educational nurses support young T1DM patients in diabetes therapy and help them accept their illness. Young adults with T1DM need support of psychologists.

## Data Availability

The original contributions presented in the study are included in the article/supplementary material. Further inquiries can be directed to the corresponding author.

## References

[1] Patterson CC Karuranga S Salpea P Saeedi P Dahlquist G Soltesz G. IDF diabetes atlas: worldwide estimates of incidence, prevalence and mortality of type 1 diabetes in children and adolescents: results from the international diabetes federation diabetes atlas, 9th edition. Diabetes Res Clin Practice. (2019) 157:107842. doi: 10.1016/j.diabres.2019.107842, PMID: 31518658

[2] Wijk I Amsberg S Johansson UB Livheim F Toft E Anderbro T. Impact of an Acceptance and Commitment Therapy programme on HbA1c, self-management and psychosocial factors in adults with type 1 diabetes and elevated HbA1c levels: a randomised controlled trial. BMJ Open. (2023) 13:e072061. doi: 10.1136/bmjopen-2023-072061, PMID: 38101850 PMC10729111

[3] DiMeglio LA Evans-Molina C Oram RA. Type 1 diabetes. Lancet. (2018) 391:2449–62. doi: 10.1016/S0140-6736(18)31320-5, PMID: 29916386 PMC6661119

[4] Reddy M Rilstone S Cooper P Oliver NS. Type 1 diabetes in adults: supporting self management. BMJ. (2016) 352:i998. doi: 10.1136/bmj.i998, PMID: 26965473

[5] Jull J Witteman HO Ferne J Yoganathan M Stacey D. Adult-onset type 1 diabetes: A qualitative study of decision-making needs. Can J Diabetes. (2016) 40:164–9. doi: 10.1016/j.jcjd.2015.09.080, PMID: 26923335

[6] Shiel EV Hemingway S Burton K King N. Self-management of type 1 diabetes in young adults: Is it impeded by aspects of everyday life? A scoping review. Diabetes Metab Syndr. (2023) 17:102918. doi: 10.1016/j.dsx.2023.102918, PMID: 38064953

[7] Bronner MB Peeters MAC Sattoe JNT van Staa A. The impact of type 1 diabetes on young adults’ health-related quality of life. Health Qual Life Outcomes. (2020) 18:137. doi: 10.1186/s12955-020-01370-8, PMID: 32398086 PMC7218580

[8] Nettleton JA Burton AE Povey RC. No-one realises what we go through as Type 1s”: A qualitative photo-elicitation study on coping with diabetes. Diabetes Res Clin Pract. (2022) 187:109876. doi: 10.1016/j.diabres.2022.109876, PMID: 35439539

[9] Juczyński Z Ogińska –Bulik N. Skala Odczuwanego Stresu – PSS-10 In Narzędzia Pomiaru Stresu i Radzenia Sobie ze Stresem; Pracownia Testów Psychologicznych Polskiego Towarzystwa Psychologicznego: Warsaw, Poland. (2012). p. 11–22.

[10] Juczyński Z. Narzędzia Pomiaru w Promocji i Psychologii Zdrowia; Pracownia Testó́w Psychologicznych Polskiego Towarzystwa Psychologicznego: Warsaw, Poland (2012). p. 162–6.

[11] Diabetes Poland. Standards of Care in Diabetes. The position of Diabetes Poland (2025). Available online at: https://www.currenttopicsIndiabetes.com/Standards-of-Care-in-Diabetes-nThe-position-of-Diabetes-Poland-2025,203685,0,2.html (Accessed March 15, 2025).

[12] American Diabetes Association. The American Diabetes Association Releases the Standards of Care in Diabetes (2024). Available online at: https://diabetes.org/newsroom/press-releases/american-diabetes-association-releases-standards-care-diabetes-2024 (Accessed March 15, 2025).

[13] International Society for Pediatric and Adolescent Diabetes. ISPAD Clinical Practice Consensus Guidelines (2024). Available online at: https://www.ispad.org/resources/ispad-clinical-practice-consensus-guidelines.html (Accessed March 15, 2025).

[14] Lindholm Olinder A DeAbreu M Greene S Haugstvedt A Lange K Majaliwa ES. ISPAD Clinical Practice Consensus Guidelines 2022: Diabetes education in children and adolescents. Pediatr Diabetes. (2022) 23:1229–42. doi: 10.1111/pedi.13418, PMID: 36120721 PMC10107631

[15] Owusu BA Ofori-Boateng P Forbes A Doku DT. Knowledge of young people living with type 1 diabetes and their caregivers about its management. Nurs Open. (2023) 10:2426–38. doi: 10.1002/nop2.1498, PMID: 36448367 PMC10006669

[16] Santos FRM Bernardo V Gabbay MAL Dib SA Sigulem D. The impact of knowledge about diabetes, resilience and depression on glycemic control: a cross-sectional study among adolescents and young adults with type 1 diabetes. Diabetol Metab Syndr. (2013) 28:55. doi: 10.1186/1758-5996-5-55, PMID: 24289093 PMC3849685

[17] Mansour-Ghanaei R Joukar F Soati F Khanegha AG. Association between knowledge, locus of control and health belief with self-management, HbA1c level and number of attendances in type 1 diabetes mellitus patients. Int J Clin Exp Med. (2013) 6:470–7., PMID: 23844271 PMC3703118

[18] Cho MK Kim MY. Educational needs of people with type 1 diabetes mellitus and their parents: A cross-sectional study. Nurs Open. (2023) 10:4849–58. doi: 10.1002/nop2.1737, PMID: 37043406 PMC10277413

[19] Balfe M Doyle F Smith D Sreenan S Brugha R Hevey D. What’s distressing about having type 1 diabetes? A qualitative study of young adults’ perspectives. BMC Endocr Disord. (2013) 13:25. doi: 10.1186/1472-6823-13-25, PMID: 23885644 PMC3733731

[20] Cyranka K Juza A Kwiendacz H Nabrdalik K Gumprecht J Małecki M. Evaluation of psychological resources of young adults with type 1 diabetes mellitus during the transition from pediatric to adult diabetes clinics: multicenter cross-sectional study. JMIR Form Res. (2023) 7:e46513. doi: 10.2196/46513, PMID: 37247225 PMC10262019

[21] Badura Brzoza K Główczyński P Piegza M Błachut M Sedlaczek K Nabrdalik K. Acceptance of the disease and quality of life in patients with type 1 and type 2 diabetes. Eur J Psychiatry. (2022) 36:114–9. doi: 10.1016/j.ejpsy.2021.12.001

[22] Lunkenheimer F Eckert AJ Hilgard D Köth D Kulzer B Lück U. Posttraumatic stress disorder and diabetes-related outcomes in patients with type 1 diabetes. Sci Rep. (2023) 13:1556. doi: 10.1038/s41598-023-28373-x, PMID: 36707607 PMC9883226

